# National pilot of innovative early years skill mix working in five Start for Life sites in England: study protocol

**DOI:** 10.1017/S1463423626100966

**Published:** 2026-03-26

**Authors:** Jane Barlow, Michael Fanner, Michelle Sleed, Laurie Day, Gabriella Conti, Gabriel Weber Costa

**Affiliations:** 1 Department of Social Policy and Intervention, University of Oxford, Oxford, UK; 2 Anna Freud Centre, UK; 3 Ecorys, UK; 4 Department of Economics, University College London, UK

**Keywords:** early years, health visiting, healthcare workforce, infant mental health, skill-mix, staffing

## Abstract

**Background:**

England’s Family Hubs and Start for Life (SfL) Programme Guidance recommends strengthening early years services by increasing workforce capacity and capability through innovative skill mix models. However, evidence regarding how different innovative early years skill mix workforce models operate, function, and influence outcomes remains limited. To address this gap, five local authorities in England that are existing SfL sites received funding from the Department of Health and Social Care (DHSC) to design and pilot innovative early years skill mix workforce models to enhance their Family Hub offers and better support families with children under two.

**Methods:**

The evaluation is guided by each site’s Theory of Change and uses a mixed-methods design. The study consists of five workstreams. First, pilot models will be mapped through documentary analysis, including content analysis of role descriptions and audits of workforce activities recorded in clinical diaries. Second, system-level mechanisms, facilitators, and barriers to implementation will be examined through reviews of service and management data and semi-structured interviews with key stakeholders. Third, relational structures underpinning effective practice will be explored using 75 family-level case studies and Social Network Analysis to assess professional networks and their influence on family and practitioner experiences. Fourth, impacts will be estimated using Synthetic Control Methods to assess effects on Healthy Child Programme outcomes, alongside cost and cost-benefit analyses. Finally, the broader application of skill mix working will be explored through semi-structured interviews and case studies across additional local authorities.

## Strengths and limitations

- This research will provide much needed information about whether innovative early years skill mix workforce models, being piloted in five SfL sites, are providing safe, acceptable and feasible contributions to the delivery of health visiting/0–5 public health nursing services.
- The extensive mapping and process data will provide a comprehensive picture of the innovative early years skill mix models being implemented, as well as the barriers and facilitators to their adoption. This will include the use of deductive content analysis of pilot role descriptions to explore how safety-critical architecture is distributed across the stratified pilot workforces.
- The use of Social Network Analysis will enable us to understand the patterns of interaction between different members of the pilot skill mix team and the ways in which these networks facilitate or hinder the success of such working.
- The use of Synthetic Control Methods will provide a reliable estimate of the impact of pilot skill mix working on health visiting/0–5 public health nursing within varying service delivery contexts.
- However, the piloting of the innovative early years skill mix workforce model is only operational for a short period of time, which will limit the full realisation of any impact of such working arrangements.
- Furthermore, the applicability of the Synthetic Control Method is limited by the availability and quality of data on both the treated unit and a suitable pool of untreated comparison units.

## Introduction

The ability of a health care system to provide safe, high-quality, cost-effective, and person-centred services depends on the extent to which sufficient, well-motivated, and appropriately skilled and knowledgeable personnel are able to operate within service delivery models that optimise their performance (Spooner *et al*., 2022). Over the past two decades, workforce skill mix has been introduced into health care settings (i.e. both primary and secondary in both high- and low-income settings) with the aim of addressing the lack of an available highly-skilled workforce and financial restrictions, within a wider context of demographic changes and increased demand for health care services. Such skill mix can include the introduction of new roles and/or the delegation of clinical activities to less qualified staff. This can have significant implications in terms of the extent to which the care provided continues to be safe, effective, and acceptable to both service providers and recipients, and the determination of the skill mix required should be led by identified local health needs and underpinned by a robust workforce plan.

Recent policy changes within England’s early years sector involving the development of Family Hubs and the Start for Life (SfL) Programme (DHSE and DfE, 2022) recommends increasing workforce capacity and capability through the use of new workforce models that incorporate skill mix and facilitate closer working across child and family welfare professions. These recommendations are based on the current guidance for local government, the National Health Service (NHS), integrated care boards and other partners, which supports practitioners in terms of the local interpretation and implementation of the Healthy Child Programme (HCP) framework, and which includes limited anticipatory advice for deciding a local workforce stratification. Such skill mix working within the Family Hubs and SfL programmes involves the reallocation of clinical activities that were undertaken by the most skilled and resource intensive professionals being allocated to more junior (i.e. lower qualified) colleagues. Such skill mix working in the early years sector thereby involves some of the clinical activities that were undertaken by health visitors being undertaken by public health staff nurses, community nursery nurses and other support staff, such that families can access the support that they need from this more diverse workforce in a variety of settings as specified in the guidance (e.g. home visits, well baby clinics, at their General Practitioner (GP) practice and in children’s centres). Contextually, a substantial expansion in skill mix has occurred alongside a 40% reduction in health visitor workforce numbers since the last national health visitor recruitment programme (2010–2015) (Fanner *et al.*, 2025).

Irrespective of the setting in which skill mix is used, its introduction is now recognised to potentially pose a range of unintended or unknown complications in terms of usual practice, including no mandated government guidance in terms of what skill mix should consist of and involve, fragmented commissioning of health visiting/0–5 public health nursing services to a range of local service providers, the use of piecemeal changes, task drift, and threat to professional identities (Dubois and Singh, 2009). Furthermore, while skill mix changes have been identified as being key to responding to changing patient needs (e.g. patients with chronic conditions and multimorbidity), unequal access to services (e.g. for vulnerable groups), skill gaps (e.g. long-term and palliative care), and changes among the health workforce (e.g. shortages and maldistribution) (Kulhman and Jong, 2018), evidence regarding their effectiveness in addressing these issues in the varying configurations and stratifications of practitioners within the health visiting workforce remains sparse (Conti *et al.*, 2025). Specifically, there is currently limited evidence concerning the impact of such skill mix working in terms of the experiences of service providers (i.e. changes in scope of practice with increased complexity) or users (i.e. experience of services by non-traditional practitioners), or indeed service-use by underrepresented demographic groups; provision of interventions within the six Early Years High Impact Areas, completion of the mandated health visitor contacts/reviews, safeguarding, or quality of the health visiting/0–5 public health nursing service.

There is a diversity of service delivery contexts within health visiting/0–5 public health nursing in England which is influenced by: the wide-ranging scope of the HCP (Elearning for Healthcare, 2022); the unwarranted variation in local commissioning decision-making (Fanner *et al*., 2025); the variety of service providers (Whittaker *et al.*, 2021); and the high needs of families in a context of budgetary restraint and scarce resources (including a shortage of qualified health visitors) (Harron *et al.*, 2024). As a result of this, there are a number of barriers to effective implementation including long-standing recruitment challenges within the early years sector (particularly that of lower graded practitioners), long-standing integrative working challenges especially within a mixed economy of service providers (i.e. NHS, local authority (LA) and the private, voluntary and independent sectors), and new ways of developing skill mix practice in the context of traditionally and locally understood ways of utilising such practitioners (which may affect referrals into the pilot innovative early years skill mix workforce models). To mitigate potential challenges, each pilot site has developed a Theory of Change (ToC) to assist in the implementation of pilot innovative early years skill mix workforce models with available support from both the Department of Health and Social Care (DHSC) and the evaluation teams.

This research aims to increase understanding about skill mix working in the five SfL sites that received additional funding, in addition to providing wider contextual information across the remaining 70 SfL sites. The success of the pilot innovative early years skill mix workforce models will be assessed in terms of its acceptability, feasibility and impact.

## Methods

### Overarching evaluation design and framework

**Figure 1.**
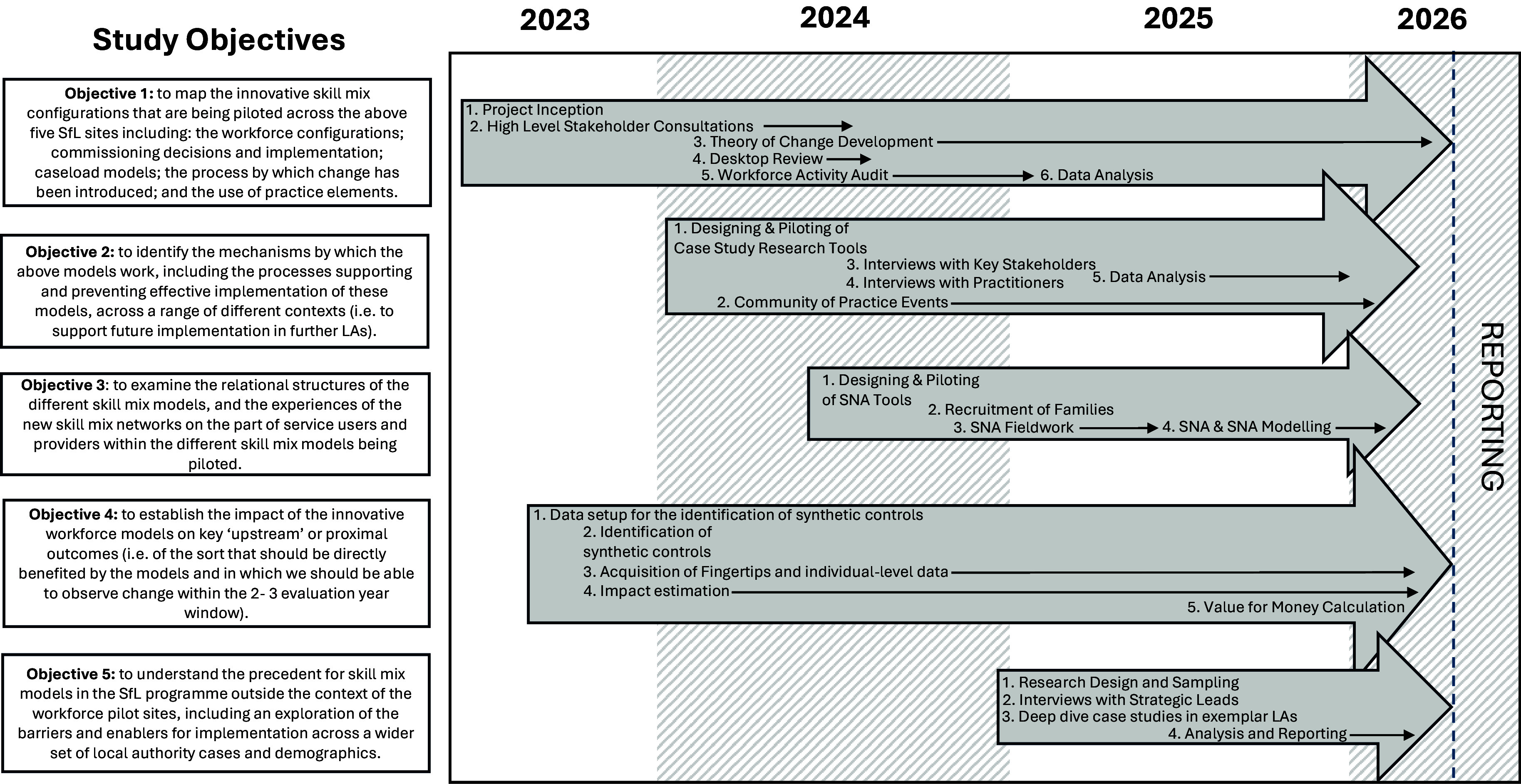
Study Flowchart.

This study comprises a mixed-methods design involving the analysis of a range of quantitative and qualitative data from both primary and secondary sources, and underpinned by the ToCs for each pilot site. We will use Social Network Analysis (SNA) to address the ways in which the networks, that are established within the skill mix team and the wider workforce, contribute to the success (or lack of it) of skill mix working within the context of the SfL Programme. Figure 1 illustrates a simplified flowchart of the study objectives and methodologies across the study timeline.

### Study objectives

The five key objectives are as follows:

Objective 1: to map the innovative skill mix configurations that are being piloted across the above five SfL sites including: the workforce configurations; commissioning decisions and implementation; caseload models; the process by which change has been introduced; and the use of practice elements (a separate qualitative process evaluation is concurrently taking place that specifically focuses on the practice elements approach within this workforce pilot (Fanner *et al.,* 2024)).^1^

Objective 2: to identify the mechanisms by which the above models work, including the processes supporting and preventing effective implementation of these models, across a range of different contexts (i.e. to support future implementation in further LAs).

Objective 3: to examine the relational structures^2^ of the different skill mix models, and the experiences of service users and providers within the different skill mix models being piloted.

Objective 4: to establish the impact of the innovative workforce models on key ‘upstream’ or proximal outcomes (i.e. of the sort that should be directly benefited by the models and in which we should be able to observe change within the 2–3 evaluation year window).

Objective 5: to understand the precedent for skill mix models in the SfL programme outside the context of the workforce pilot sites, including an exploration of the barriers and enablers for implementation across a wider set of local authority (LA) cases and demographics.

### Sample

The primary focus of the research will be on the five pilot sites that applied for and were awarded additional funding as part of the SfL Programme, to implement skill mix innovations (Objectives 1 to 3). For the impact analysis using the Synthetic Control Method (Objective 4), the remaining 70 funded SfL sites will be used for the purpose of counterfactual data, whereby a weighted average of the control sites is used as a counterfactual (a synthetic control) for each treated site.

The evaluators will also conduct data collection in SfL sites without additional skill mix funding, to establish the precedent for skill mix in the context of the wider SfL programme (implementation evaluation) (Objective 5). This will include the addition of a number of skill mix items within the survey of local strategic leads in all SfL funded sites, which is being carried out as part of the National Evaluation of SfL (Barlow *et al*., 2025); targeted semi-structured interviews with skill mix managers or commissioners in a sub-sample of 15–20 LAs without additional skill mix funding, and case study research with skill mix practitioners in four such LAs. The latter cases will be selected on the basis of having an established local skill mix model, including a bespoke training package; more than one stratification below a health visitor level, and skill mix roles focused on a range of clinical activities beyond the completion of mandated health visitor contacts/reviews.

### Data collection and sources

#### Primary data

*i. Baseline mapping of models across pilot sites* (Objective 1): The key data sources for the baseline and system change stages of the research will be the site application and related documents depicting the skill mix changes being introduced and piloted for each of the sites, and Management Information (MI) including delivery plans and associated KPIs/metrics. We will populate the Reach, Effectiveness, Adoption, Implementation and Maintenance (RE-AIM) framework matrixes for each LA with the demographic and service use information, e.g. HCP use by under-represented populations, and corresponding outcomes data (e.g. workforce retention rates, caseload size, completion rates for the five mandated health visitor contacts/reviews and additional contact points). We will also review role descriptions and person specifications of each of the clinically facing roles within the five pilot sites through a planned deductive content analysis (McKibben *et al.*, 2022). The baseline mapping will occur between February 2024 and May 2024.

We will conduct a cross-sectional audit of workforce resources in the five pilot sites, with the aim of better understanding the allocation of daily and weekly clinical and non-clinical activities of skill mix practitioners within the innovative workforce models, and adjacent universal services. This audit will involve the extraction of anonymised workforce activity data from practitioners’ clinical diaries, with a focus on process rather than outcomes. The audit will be conducted between January 2024 and March 2026. The de-identified data from this audit will be shared with the evaluation team through a data sharing agreement.

We will also conduct semi-structured interviews with at least two representative members of each level of the skill mix team within the five pilot sites (*n* = up to 50 interviews in total). The focus of the interviews will be to establish a bottom-up picture of how the innovative skill mix work is progressing, and views about what could be done to improve its functioning. We will also survey all staff in the skill mix teams within the five pilot sites to ascertain: qualifications, training, experience, skills and work profile as part of the practice elements approach within this workforce pilot (Fanner *et al.,* 2024). We will seek further information about caseload profiles including: number of families on each caseload; number of children with identified special needs; number of children with child protection or child in need plans; identification of GP attached children; identification of geographical families, for example, those registered with GPs outside the area including those in temporary housing; current screening practice for children aged eight months, two years and 42 months, and immunisation practice, clinic and group sessions. For further perspective, we will also interview (i) commissioners about their aspirations when commissioning the health visiting/0–5 public health nursing service specification and to identify the criteria that they used to make their judgements about the staff needed to meet local need, and (ii) pre-existing caseload holders and supervisors on their experiences of the innovative skill mix arrangements being piloted. Interviews will take place between October 2024 and March 2025 following UK Integrated Research Application System (IRAS) approval. There are no planned follow-up interviews.

*ii. Measuring system level change:* Objective 2 is focused on the implementation of the local skill mix models, including the steps taken to recruit, train and equip the local workforce, how families are identified, engaged and supported, and service pathways. This objective includes both process and outcomes dimensions, with a focus on the system level. The mapping exercise described in Objective 1 will constitute a baseline for the later fieldwork, and each subsequent data collection stage will include semi-structured interviews with senior managers and commissioners overseeing local skill mix implementation to establish the overall strategic context, updates to the model, links to wider Family Hub and SfL infrastructure workforce development and service transformation. Interviews will take place between October 2024 and March 2025 following IRAS approval. There are no planned follow-up interviews.

iii. *Case studies* will be carried out to address Objective 3, with each case being defined as a ‘family unit’. Within each of the five pilot sites, we will recruit 15 families (*n* = 75), comprised of one or more parents or caregivers and their infant/s. Families will be eligible to take part if they received services from the skill mix team, and they had at least one infant aged 0 to 24 months of age at the point of receiving these services. The methods for identifying and recruiting participants have been developed through consultation with the project parent-carer advisory group (PCAG). Referrals will be made by caseload holding clinicians in each pilot site. They will be asked to consecutively invite all families who have most recently received the package of services that they are piloting within their local models and for whom the work is complete. To ensure representation of vulnerable and seldom-heard participants in the sample, several measures to ensure accessibility will be taken. These include £50 vouchers for participant time, flexibility in the format of the interview (online or in-person), funding for a device, data, or transport, and interpretation services, as required. Data will be collected through semi-structured interviews with families and professionals in the network around them. As we will be analysing the networks around individual families (‘ego-centric networks’), the first stage of data collection will be from a parent or carer in each family. These interviews will be conducted using Network Canvas. The interview protocol will determine the support networks around each family, and will also include in-depth open-ended questions about the service users’ experiences of the services they have received. Follow-up questions will be asked as appropriate to the conversation. All interviews will be recorded and transcribed and framework analysis undertaken to obtain a detailed and in-depth perspective from each family, across all families within each LA as well as across the complete dataset of families. For a subsample of five families per pilot site (*n* = 25), snowballing sampling methods will be used to follow-up the named professionals identified by the families (direct network). These professionals will be asked about their relationship and contact with the family, and also about the network around them that directly or indirectly enabled them to support the family (indirect network). Brief interviews with these professionals about their experiences of this way of working will also be carried out. In this way, we will develop a picture of the professionals and the relational, communication and supervision structures that contribute ultimately to the families’ experiences and access to support and information. The SNA protocol development, recruitment of families and SNA fieldwork will occur between May 2024 and March 2025 following IRAS approval. There are no planned follow-up interviews with families.

*iv. Additional primary data collection* will be conducted to address Objective 5 in terms of understanding the precedent for skill mix activity across the wider set of (70) LAs within the SfL programme. This workstream will aim to establish the barriers and enablers to the design and implementation of local skill mix models across a range of different contexts and demographics, and to understand the specific ways in which skill mix has been initiated and/or adapted to enhance the delivery of local Family Hubs and SfL services and support. A set of screening questions will be included within the wider survey of SfL local strategic leads, which is scheduled for completion by all funded LAs as part of the national evaluation of SfL (Barlow *et al.,* 2025). This screening exercise will aim to establish which LAs are making use of skill mix models in conjunction with their local SfL offer; to gather the information required to inform an initial selection of LAs according to their correspondence with the sampling criteria (having a bespoke training package; more than one stratification below a health visitor level, and skill mix roles focused on a range of clinical activities beyond the completion of mandated health visitor contacts/reviews), and to obtain consent for re-contact to gather supplementary information to inform sampling.

The screening data from the survey will guide the selection of a sub-sample of 15–20 LAs for semi-structured telephone interviews with managers or commissioners holding a lead role for skill mix. These will seek to determine the origins and aims of the local skill mix model, key characteristics, and lessons learned from implementation, as well as establishing how outcomes are captured and measured within the context of service implementation. These interviews will inform the selection of (*n* = 3–4) LAs for case study research. Cases will be selected on the basis of providing more established models that illustrate the potential utility of skill mix when embedded within local service frameworks, alongside the evidence gathered from early implementation within the five pilot sites. Each case study will include semi-structured interviews with staff involved in planning and commissioning the local skill mix model (*n* = 1–2); semi-structured interviews and/or an online focus-group with skill mix practitioners (6–8); and reviews of key documentation (e.g. health visiting/0–5 public health nursing service specifications). These data will be coded for thematic analysis, alongside qualitative evidence gathered from the pilot sites. This additional primary data collection will occur between January 2025 and June 2025. There are no planned follow-up interviews.

#### Secondary data sources

To assess impacts we will use micro data both at the level of LA and at individual level, in order to maximise coverage and minimise possible delays in data acquisition. The main source of LA-level data will be Fingertips Child and Maternal Health Data available from the Office for Health Improvement and Disparities (OHID) public website, which includes key indicators such as vaccination coverage rates and health visiting metrics (completion rates of mandated reviews, percentage of face-to-face visits). Individual-level data from the Education & Child Health Insights from Linked Data (ECHILD) database will also be used (Ramzan *et al.*, 2023). The ECHILD contains linked administrative data from NHS hospitals, as well as education and social care services, covering all children in England. It includes data from: Community Services Data Set (CSDS), which records information such as mandated health visitor contacts/reviews and referrals to community health services; Maternity Services Data Set (MSDS), which includes variables such as Apgar score (a standardised assessment of a infant’s status immediately after birth), birth weight, and first feed status; Mental Health Services Data Set (MHSDS), which includes information on secondary care for parents and children, for mental health and learning disabilities (such as referral, reason, and treatment); Hospital Episode Statistics (HES), which includes information admitted patient care, critical care, accidents and emergencies, outpatient care and mortality records; Children In Need (CIN)/Children Looked After (CLA), with records of all children referred to children’s social care services, including those looked after by a LA. It is worth noting that all indicators considered will be measured in the short-term, given the short-term nature of the funding for the pilot models and their recent implementation. Secondary data sources will be collected (with periodic refresh) between December 2023 and April 2026.

### Data analysis

A range of methods will be used to analyse the data that has been collected for each of the five study objectives:

*Documentary data analysis (Objective 1):* Data from all documents will be used to provide a narrative, i.e. qualitative rich, descriptions of each pilot model, with the use of tables and organograms to illustrate model development using two approaches: (1) through individual case studies, (2) a meta-level overview of model development across all five pilot sites, and (3) a planned deductive content analysis (DCA) (McKibben *et al.*, 2022) of the role descriptions of each of the clinically facing roles within the five pilot sites on NVivo. The DCA will better enable us to understand how safety-critical architecture (e.g. clinical accountabilities and responsibilities) is distributed across the pilot workforce stratification, individual role contributions and responsibilities within the family journey (e.g. assessment, care planning, delivery of care, review/evaluating care) and specific role alignment with the practice elements approach modular content (see Fanner *et al.,* 2024). This analysis will occur between February 2024 and March 2026, with a large proportion completed by September 2024 for the first year interim report to the funder.

#### Qualitative data analysis

*Framework Analysis (Objective 1, 2, and 3) –* Interview data with parents/carers and professionals about their experiences will be analysed using a Framework analysis (Parkinson *et al.*, 2016). The analysis will be conducted using NVivo. This will involve systematically analysing qualitative data through a structured yet flexible approach. Two researchers will conduct the analyses. A thematic framework will be developed, combining predefined themes and those emerging from the data, which will guide the open coding process. Finally, data will be organised into a matrix for charting, enabling patterns, connections, and key insights to be interpreted. To ensure the rigour of the analysis, a subset of transcripts will be independently coded by both researchers, who will meet regularly to discuss the credibility of emerging results and refine the coding framework as needed. These findings will also be interpreted alongside the SNA data, thus enabling us to understand how the workforce structures relate to families’ experiences of services. Data will be interpreted at the individual level (case study) and LA (pilot model) level. This data analysis will occur between April 2025 and September 2025.

Framework analysis will also be utilised for the data gathered from skill mix practitioners and key stakeholders in the wider set of LAs (Objective 5). This strand will be grounded in the framework developed within the pilot sites, ensuring consistency in terminology and pre-defined themes. The framework will undergo further iteration as primary data is gathered and coded in NVivo. This will start with the addition of descriptors corresponding with the skill mix models identified through the screening exercise and the semi-structured interviews with managers and commissioners. The coding and analysis will be further deepened with the incorporation of data from the qualitative interviews with skill mix practitioners within case study LAs. Data analysis will take place both within, and between, local cases. The researchers will seek to establish the factors that have enabled or inhibited skill mix development, considering the different approaches to workforce stratification, training and supervision, and different local demographics. They will further draw on the data to compare and contrast how skill mix has been initiated and/or adapted to enhance the delivery of local Family Hubs and SfL services and support. This data analysis will occur between August 2025 and September 2025.

*SNA (Objective 3)* will be conducted using R statistical software. Visual sociograms will be used to assess the structure and characteristics of the support networks around the families. We will calculate metrics such as: the number of support ties, diversity of roles among ties, and evaluation of how supportive or not each tie was. These will be used to understand between-family diversity in support networks, and assess if these structures are related to overall perceived satisfaction with care. Multilevel models with tie satisfaction as the level 1 outcome, and family at level 2 will assess between family variation in satisfaction with support received, and variation in role type modelled as a fixed effect. Where data permits, we will consider using a cross-classified multilevel model to account for multiple ratings of the same role type. We will make a qualitative comparison of the visual structure of networks for participants with the lowest and highest general satisfaction with care, and consider similar visual analysis by other important family characteristics. These models will help to characterize the amount of variability in family experiences, identify if there are any systemic issues which are regularly reported as more or less positive experiences. These will be supplemented with additional explanatory information from the qualitative transcripts, providing key insights into areas for improvement, and also identification of areas of promising practice. This data analysis will occur approximately between September 2025 and January 2026.

#### Quantitative data analysis

Descriptive statistical analysis will be used to explore the de-identified data from the cross-sectional audit of workforce resources in the five pilot sites.

*Impact analysis (Objective 4):* We will work with LA- and individual-level data key to measure outcome indicators, such as those outlined in the *Secondary Data* sources section above. We will estimate the impacts of the workforce models using a Synthetic Control Method (SCM) (Abadie and Gardeazabal 2003; Abadie *et al.*, 2010), testing whether the models causally affect outcomes, e.g. completion rates of mandated health visitor contacts/reviews. Synthetic controls will be constructed as weighted averages of untreated units to match on pre-intervention outcomes levels and trends, and factors which could affect them (technically, to minimise the mean square prediction error). The SCM allows us to obtain separate estimates of causal effects for each of the five treated sites, which is particularly useful in the context of the innovative early years skill mix models, given the variability of implementation across sites. We will also examine whether effects differ by relevant groups (e.g. socioeconomic status, ethnicity). To account for the fact that applicability of the SCM hinges on the quality of the data available, we will triangulate data sources whenever possible (e.g. HCP completion rates can be sourced from the CSDS and from the health visitor service delivery metrics published quarterly by the OHID), and potentially utilise novel approaches that facilitate SCM estimation in the presence of multiple outcomes, even when there is some missing data in the pre-treatment period (Sun *et al*., 2025). We will also leverage recent advances in SCM, which will enable us to account for imperfect pre-treatment fit (Ben-Michael *et al.*, 2021; Ferman and Pinto 2021) and to perform a finer assignment of weights to untreated units (Abadie and L’Hour, 2021; Kellogg *et al.*, 2021). Following best practice, we will perform extensive robustness tests and assess the sensitivity of the results to the use of alternative methods, such as Difference-in-Differences (Ferman and Pinto, 2021) and Synthetic Differences-in-Differences (Arkhangelsky *et al*., 2021). Furthermore, we can also explore estimating counterfactuals by measuring the outcomes of children unlikely to have benefitted from the pilots, based on observed characteristics such as age or distance to a family hub. This analysis will take place throughout the period between December 2023 and April 2026.

*Cost-Benefit Analysis (Objective 4):* We will compute the costs incurred to implement each of the five workforce pilots (for example, recruiting and training new personnel, performing additional health contacts/visits, etc.), and evaluate them vis-à-vis the monetised benefits estimated in the impact evaluation. We will work with DHSC to put systems in place so that the service-level data being collected is fit for purpose. We will combine the impact estimates obtained with the best available sources for costing, such as the NHS National Cost Collection for the costs of NHS services. We will follow NICE guidelines (including recommended discount rate and performing extensive sensitivity analysis) and compute the costs and benefits from the perspective of those providing and using the services. Although potential future benefits that exceed the timing of the project cannot be directly quantified, we will account for long-term returns in the Cost-Benefit Analysis by using ‘surrogate index’ methods (Athey *et al.*, 2024), informed by the existing literature. Given the inherent uncertainties, we will also perform sensitivity analysis to account for different scenarios of future benefits. This analysis will take place between February 2026 and April 2026.

## Discussion

Overall, the data within this evaluation will reflect a relatively short period of time in operation due to the nature and length of the government funding. It is not intended for each of the pilot models to be generalisable, rather that specific learning can be achieved with regard to the safe, acceptable and feasible implementation of innovative skill mix workforce modelling when addressing differing local needs, systems and workforces within each LA in the delivery of health visiting services. This will mean variability across each pilot site is expected. We have planned for additional data collection in LAs external to the pilot model but within the wider Family Hubs and SfL programme. This will bring additional skill mix workforce models within the scope of the analysis to understand what works in a wider range of contexts, including where skill mix models have been established for longer.

## Ethics and dissemination

Ethics committee approval has been secured from IRAS (24/LO/0371) and the University of Oxford, the latter of whom will also sponsor the study. The main ethical considerations will be concerned with a) participant burdens and/or risks b) the physical and psychological safety of the study researchers; and c) consent, data protection/ confidentiality. We will ensure that all researchers abide by the recognised GDPR, International Council for Harmonisation - Good Clinical Practice (ICH-GCP), and local safeguarding procedures, and that they have been appropriately trained and certified.

The final outputs, engagement, and dissemination plans will be co-produced with families, participating sites and the DHSC, and will aim to identify key audiences and channels, and leverage maximum research impact through a range of methods including policy workshops, practice guidelines and parent-friendly information. We will also connect with networks established via the National Centre for Family Hubs and Anna Freud Centre’s Early Years in Mind network, and the Supporting Early Minds Research Network (Oxford Health NIHR BRC).

Participants and the wider population will be kept informed about progress and the findings throughout the duration of the study using a range of accessible methods (i.e. newsletters, leaflets, website, blogs, and social media) in relevant different languages and formats. We will support our PCAG to perform a role as Learning Champions, raising awareness of the evaluation within the SfL LAs, and disseminating more widely via parenting networks as multipliers. Through this approach we will aim to boost awareness and reach with culturally and linguistically diverse communities and with families who may not be accessing SfL services.

The findings of the research will be disseminated to relevant audiences using conference presentations, and publications in academic, policy and methods journals. We will also hold at least one roundtable session, bringing together civil servants, professional associations, third sector partners, and parent and carer representatives to consider the implications for policy and practice.

## Footnotes

1
Practice elements refer to the essential components for a successful implementation of a policy intervention. Practice elements are identified as the common elements or practices frequently shared among similar policy interventions, which are more likely to contribute to positive outcomes.

2
Relational structures refer to the social ties between individuals within a social system, in this case the direct and indirect professional system centred around the service-user, and which act as a system for the provision of relevant information, services and support.
